# Assessment of Physicians’ Knowledge and Awareness About the Hazards of Radiological Examinations on the Health of Their Patients at a Tertiary Care Hospital, Riyadh, Saudi Arabia

**DOI:** 10.7759/cureus.27479

**Published:** 2022-07-30

**Authors:** Raneem H Najjar, Azouf M Alsulaiman, Jumanah S Alraddadi, Nehal H Alrohaimi, Bsma A Algarni, Assad M Al-Arafa, Reem A Alsubait

**Affiliations:** 1 Family Medicine, King Saud Bin Abdulaziz University for Health Sciences College of Medicine, Riyadh, SAU; 2 Family Medicine, King Abdulaziz Medical City Riyadh, Riyadh, SAU; 3 Medical Education, King Saud Bin Abdulaziz University for Health Sciences College of Medicine, Riyadh, SAU

**Keywords:** survey, kap, practice, attitude, hazards, knowledge, alara, awareness, safety, radiation

## Abstract

Background

One of the vital tools in diagnosing a variety of medical conditions is through radiological examinations which can lead to severe biological effects if precautions are not taken. To limit the harmful effects, as low as reasonably achievable (ALARA) was implemented. ALARA aims to minimize the time, increase the distance, and promote the use of protective shielding.

Method

The cross-sectional study included 454 physicians in King Abdulaziz Medical City (KAMC) and King Abdullah Specialist Children’s Hospital (KASCH), Riyadh, Saudi Arabia. The study assessed physicians’ knowledge and awareness about the hazards of radiological examinations on their patients’ health using a self-administered questionnaire to measure knowledge, attitude, and practice (KAP). KAP was compared with the sociodemographic characteristics using the Mann-Whitney Z-test as well as Kruskal Wallis H-test.

Results

Out of 454 physicians, males exceeded the females (61.7% vs 38.3%) with nearly three-quarters (72.5%) working in King Abdulaziz Medical City. The most commonly mentioned specialty was internal medicine, while the least common specialty was orthopedics. Based on a cutoff point of 60%, it was revealed that poor knowledge was observed in 70.5% of physicians. With regards to attitude, 65.2% of physicians had a positive attitude. For practices, 49.8% had poor practices while 50.2% had good practices. The mean scores for knowledge, attitude and practice were 9.19 (SD 7.03) out of 23 points, 1.89 (SD 1.06) out of 3 points, and 5.43 (SD 1.67) out of 8 points, respectively.

Conclusion

In conclusion, poor knowledge, practice, and positive attitude were detected among physicians. However, our study was limited by the use of a self-administered online questionnaire.

## Introduction

Nowadays and over the years, the use of imaging technology in the medical field has been vital to confirm, accurately assess, and document the development of many diseases, as well as assess the effectiveness of treatment [1,2]. CT and X-ray use ionizing radiation, which is the energy produced from natural or synthetic sources. Compared to non-ionizing radiation, ionizing radiation has the capacity to induce damage to living tissue by causing chemical changes in cells [3,4]. To promote public health, as low as reasonably achievable (ALARA) principle should be applied, which means that the radiation dose should be kept as low as possible. Moreover, minimizing the time, increasing the distance, and using protective shielding are the three main components of ALARA [5-7]. If precautions are not taken, severe biological effects can occur due to radiation exposure. These effects can be divided into deterministic and stochastic effects. Deterministic effects have a certain threshold dose. If the threshold dose is exceeded, it may lead to deterministic effects such as hair loss and skin burns. On the other hand, stochastic effects have no threshold. This makes the risk of side effects like cancer and teratogenic effects equal in low and high doses [8-10].

The risk of radiation exposure is greatly dependent on the level of knowledge and awareness of the health care providers [11]. Based on a systematic literature review that included more than 20 articles, physicians and patients showed inadequate knowledge regarding the associated cancer risk and radiation dose with CT [12]. Furthermore, according to Korean surveys conducted from 2011 to 2016, 61% of pain physicians who participated in the study did not receive radiation safety education. However, there was no difference between the practice of physicians who received radiation safety education and those who did not, and this showed the huge gap between the existing knowledge and the related practice [13]. In Saudi Arabia, a study was conducted in King Khalid University Hospital and King Fahad Medical Hospital in Riyadh which included 157 physicians. It was revealed that 58.6% of participants lacked knowledge about radiation dose for many common radiological examinations. Interestingly, there was no variation in the knowledge among radiologists and other physicians [14]. Another study conducted in 20 cities in Saudi Arabia with more than 450 physicians showed that about 30% of the participants have received education about radiation protection. Moreover, all these results clearly demonstrated the lack of knowledge and awareness physicians have. As a result, this leads to radiation abuse and might subject the patient’s health to a potential risk of cancer [15]. In Saudi Arabia, a number of health care facilities showed a lack of essential radiation protection equipment such as lead glasses and shields [16]. Consequently, further studies should be conducted to assess the level of protection used in each hospital to guarantee that the necessary precautions are taken.

Since the risk of radiation when safety precautions are not taken affects both the patients and health care providers who are involved in the procedure, this study aimed to assess physicians’ knowledge and awareness about the hazards of radiological examinations on the health of their patients at National Guard Hospital, Riyadh, Saudi Arabia [5]. Moreover, an important aspect of this study was to evaluate the awareness of physicians, compare physicians’ backgrounds with the results, and investigate whether the physicians’ knowledge is being applied in their practice. Our aim is to improve the quality of education. If a lack of knowledge was found or if the physician’s knowledge was insufficient, then this may indicate poor education and inadequate training in radiation protection.

## Materials and methods

Study design, area and settings

A cross-sectional study design was conducted since the study only focused on the population’s knowledge, attitude, and practice (KAP) for a specific period. This design was used to assess the KAP of physicians at the National Guard Hospital which has the largest imaging facility in the Middle East. There are several specialist hospitals and health care centers in NGHA to meet the increased demands for health care services, one of which is King Abdulaziz Medical City and King Abdullah Specialist Children’s Hospital. The significance of these two health care centers is demonstrated in their massive bed capacity. King Abdulaziz Medical City (KAMC) accounts for up to 1500 beds, and up to 600 inpatient single rooms in King Abdullah Specialist Children’s Hospital [17,18].

Identification of study participants

Our study included 454 physicians who worked in King Abdullah Specialist Children’s Hospital, Riyadh, Saudi Arabia, in addition to physicians who work in King Abdulaziz Medical City, Riyadh, Saudi Arabia. In addition to excluding interns and students, we included all physicians and excluded participants who were not present or unavailable while we were collecting our data. Our primary outcome variable was the level of knowledge, attitude, and practice which are categorical variables. The data was collected from January to July 2021. According to a study that was conducted in Palestine in 2012, the prevalence of poor knowledge about radiation risk exposure was high. The study also showed a gap of knowledge among 163 physicians that answered a questionnaire. Interestingly, 98.2% of the respondents showed a lack of awareness and knowledge about radiation safety [19]. Another study that emphasized the prevalence locally in Saudi Arabia and included more than 450 physicians showed that about 73% of the participants have not received education about radiation protection [15].

Data collection process

Data was collected through an online survey along with the consent form. It was distributed via emails to all physicians who work at King Abdullah Specialist Children’s Hospital and at King Abdulaziz Medical City, Riyadh, Saudi Arabia. A questionnaire developed by Hamarsheh and Ahmead was used in this study as shown in the Appendix (Table 6) [19]. The questionnaire consists of two sections. The first section includes six questions about sociodemographic variables such as workplace, sex, and occupation. Section 2 consists of knowledge, attitude, and practices about the hazards of radiological examination which were measured using items 15, 3, and 2 of the questionnaires, respectively. For the knowledge calculation, we identified the correct answers (Q1-10) and we coded with 1 while the incorrect answers were coded with 0. The remaining five items were placed on a 4-point Likert scale categories ranging from 1 to 4. The total knowledge score was calculated by adding all 15 items and a score range from 1 to 23 was generated which indicates that the higher the score, the higher the knowledge of the hazards of radiological examination. Regarding attitude, it consisted of three-item questionnaires, where “yes” was coded as 1 and “no” was coded as 0. The total attitude score was obtained by adding all three items. A possible score ranging from 0 to 3 was generated, indicating that the higher the score the higher the attitude toward the hazards of radiological examination. Finally, for practices, it consisted of two-item questionnaires, where “never” was coded as 1, “rarely” was coded as 2, “sometimes” was coded as 3, and “often” was coded as 4. The overall practice score was calculated by adding two questions and a score range from 1 to 8 was generated, where the higher the score the higher the practices toward the hazards of radiological examination. Based on a cut-off point of 60%, participants' knowledge, attitude and practice were classified as poor/negative level if the score is below and good/positive level if the score is above this percentage.

Data analysis

Data was entered using Microsoft Excel (Microsoft® Corp., Redmond, WA), and all data analyses were carried out using Statistical Packages for Software Sciences (SPSS) version 26 (IBM Corp., Armonk, NY). Descriptive statistics were presented using frequencies, percentages, mean and standard deviation. The knowledge, attitude, and practice scores follow abnormal distributions. Thus, non-parametric tests were applied. The KAP scores were compared with the sociodemographic characteristics using Mann-Whitney Z-test as well as Kruskal Wallis H-test. Normality tests were performed using Shapiro-Wilk test. The outcome of the scores was classified into two categories with a cut-off point of 60%. A P-value of 0.05 was considered statistically significant with a confidence interval of 95%. Informed consent was taken before filling out the questionnaire. After King Abdullah International Medical Research Center (KAIMRC) granted approval, data collection started. Confidentiality and autonomy of the health care providers were maintained throughout the research using serial numbers instead of using their names and badge numbers. Data was kept with the PI, and it was stored in a private file in a computer.

## Results

This study included 454 physicians. Table 1 described the socio-demographic characteristics of the physicians. Males outnumbered the females (61.7% vs 38.3%) by nearly three-quarters (72.5%) working in King Abdulaziz Medical City. Resident physicians constitute 59.7% while consultants constitute 26%. The most commonly mentioned specialty was medicine (18.1%), while the least specialty was orthopedics (2%).

**Table 1:** Socio-demographic characteristics of the physicians (n=454)

Study variables	N (%)
Gender	
Male	280 (61.7%)
Female	174 (38.3%)
Workplace	
King Abdulaziz Medical City	329 (72.5%)
King Abdullah Specialist Children’s Hospital	125 (27.5%)
Occupation	
Consultant	118 (26.0%)
Resident	271 (59.7%)
Other	65 (14.3%)
Specialty	
Medicine	82 (18.1%)
Pediatrics	69 (15.2%)
Family medicine	58 (12.8%)
Emergency	50 (11.0%)
Surgery	43 (09.5%)
Anesthesia	33 (07.3%)
Radiology	33 (07.3%)
Gynecology	20 (04.4%)
Orthopedics	09 (02.0%)
Others	57 (12.6%)
Graduation place	
European country/United States	36 (07.9%)
Arab country	398 (87.7%)
Other	20 (04.4%)
Years of clinical practice	
<5 years	267 (58.8%)
5 – 10 years	97 (21.4%)
11 – 20 years	46 (10.1%)
>20 years	44 (09.7%)

In the assessment of physicians’ knowledge about the hazards of radiological examination as seen in Table 2, we have learned that the prevalence of physicians, who were aware of ALARA principle, was 31.7% while the prevalence of physicians, who knew published articles on radiation hazards, was 28.6%. Likewise, the proportion of physicians, who knew about FDA medical X-ray listings related to carcinogen, was 54.4%. Furthermore, 35.7% of the physicians were correct that the radiation dose to the patient from a multi-slice CT scanner was higher than a single-slice helical scanner. The knowledge of the physicians regarding the percentages of total ionizing radiation, where the general public has been exposed, was poor, as only 15% were able to identify the correct answer, which is from 15% to 30%. Similarly, their knowledge about the recommended patient dose limit for medical radiation was also suboptimal, as only 2% were able to identify the correct answer which is no dose limit. Moreover, 31.1% were confident that both the prescriber and practitioner had the responsibility for protecting the patients. Additionally, the minority of them (4.8%) were correct that the number of chest X-ray equivalent to lumbar spine X-ray was 65. Also, 6.6% knew that the number of chest X-ray equivalent to abdominal CT scan was 250, and 4.6% were aware that the number of chest X-ray equivalent to barium enema was more than 250. The radiation sensitivity of lungs, bladder, gonads, kidney, and stomach was ranked from 0 to 4, where the higher the score the higher the sensitivity to radiation.

**Table 2:** Assessment of physicians’ knowledge about the hazards of radiological examinations (n=454) * Indicates correct answer. ALARA = As Low As Reasonably Achievable; FDA = Food and Drug Administration; CT = Computerized tomography; ICRP = International Commission on Radiological Protection.

Statement	N (%)
Aware of ALARA principle	
Yes *	144 (31.7%)
No	310 (68.3%)
Know any published articles on radiation hazards	
Yes *	130 (28.6%)
No	324 (71.4%)
Know about FDA listing medical X-rays as a known carcinogen	
Yes *	247 (54.4%)
No	207 (45.6%)
Think radiation dose to patient from multi-slice CT scanner is	
Higher than single-slice helical scanner *	162 (35.7%)
Lower than single-slice helical scanner	49 (10.8%)
Similar to single-slice helical scanner	35 (07.7%)
I don't know	208 (45.8%)
% of total ionizing radiation the general public is exposed to from medical radiation	
1 – 10	74 (16.3%)
15 – 30 *	68 (15.0%)
35 – 45	29 (06.4%)
60 – 75	05 (01.1%)
80 – 95	08 (01.8%)
I don’t know	270 (59.5%)
Recommended patient dose limit for medical radiation (mSv)	
100	17 (03.7%)
50	35 (07.7%)
20	27 (05.9%)
5	26 (05.7%)
0.5	52 (11.5%)
No dose limit *	09 (02.0%)
I don’t know	288 (63.4%)
ICRP recommendations for professional responsibility for protecting patients	
According to the freedom of prescription	13 (02.9%)
Prescriber, not practitioner	15 (03.3%)
Practitioner, not prescriber	13 (02.9%)
Both prescriber and practitioner *	141 (31.1%)
Don't know	272 (59.9%)
Number of chest X-ray equivalent: Lumbar spine X-ray	
<1	60 (13.2%)
10	71 (15.6%)
65 *	22 (04.8%)
120	12 (02.6%)
250	06 (01.3%)
>250	05 (01.1%)
I don’t know	278 (61.2%)
Number of chest X-ray equivalent: Abdominal CT scan	
<1	13 (02.9%)
10	32 (07.0%)
65	39 (08.6%)
120	55 (12.1%)
250 *	30 (06.6%)
>250	37 (08.1%)
I don’t know	248 (54.6%)
Number of chest X-ray equivalent: Barium Enema	
<1	19 (04.2%)
10	34 (07.5%)
65	45 (09.9%)
120	23 (05.1%)
250	17 (03.7%)
>250 *	21 (04.6%)
I don’t know	295 (65.0%)
Rank the radiation sensitivity	Mean ± SD Score
Lungs	1.46 ± 1.48
Bladder	1.38 ± 1.43
Gonads	1.98 ± 1.82
Kidney	1.29 ± 1.30
Stomach	0.93 ± 1.06

The assessment of physicians’ attitudes and practices about the hazards of radiological examination is given in Table 3. Pertaining to attitude, 57.3%, 66.5%, and 64.8% of physicians had a positive attitude toward reducing the use of routine X-ray, fluoroscopy, and CT scan, respectively. With regards to practices, 37% of the physicians practiced X-ray routinely for at least more than 75% of the time. In addition, 11.7% of the physicians ordered CT scans more often (>75% of the time), while 39.2% reported practicing CT scans sometimes (25-75% of the time).

**Table 3:** Assessment of physicians’ attitude and practices about the hazards of radiological examinations (n=454) CT = Computerized tomography

Attitude statement	N (%)
Reduce the use of routine X-ray (yes)	260 (57.3%)
Reduce the use of fluoroscopy (yes)	302 (66.5%)
Reduce the use of CT scan (yes)	294 (64.8%)
Practices statement	
How frequently do you use X-ray routinely?	
Never	49 (10.8%)
Rarely (<25% of the time)	105 (23.1%)
Sometimes (25 – 75% of the time)	128 (28.2%)
Often (>75% of the time)	168 (37.0%)
How frequently do you use CT scans routinely?	
Never	58 (12.8%)
Rarely (<25% of the time)	165 (36.3%)
Sometimes (25 – 75% of the time)	178 (39.2%)
Often (>75% of the time)	53 (11.7%)

Based on the given criteria, we evaluated the overall knowledge, attitude, and practices of physicians toward the hazards of radiological examinations. It was revealed that poor and good knowledge accounted for 70.5% and 29.5% of physicians, respectively. With regards to attitude, 34.8% had a negative attitude and 65.2% of physicians had a positive attitude. For practice, 49.8% and 50.2% physicians had poor and good practices respectively, as shown in Table 4.

**Table 4:** Descriptive statistics of knowledge, attitude, and practices (KAP) (n=454)

KAP variables	N (%)
Level of knowledge	
Poor	320 (70.5%)
Good	134 (29.5%)
Total knowledge score (mean ± SD)	9.19 ± 7.03
Level of attitude	
Negative	158 (34.8%)
Positive	296 (65.2%)
Total attitude score (mean ± SD)	1.89 ± 1.06
Level of practices	
Poor	226 (49.8%)
Good	228 (50.2%)
Total practices score (mean ± SD)	5.43 ± 1.67

We then performed Pearson correlation coefficient to determine the linear agreement between the knowledge, attitude, and practice scores. Based on the results, it was found that there was a positive correlation between the knowledge and attitude scores (r=0.100), while the correlation between the knowledge and practice scores as well as the attitude and practice scores did not reach statistical significance (p>0.05).

A non-parametric test was performed (Table 5) to determine the differences in the score of the knowledge, attitude, and practices in relation to the socio-demographic characteristics of physicians. The knowledge score of male physicians was significantly better than female physicians (Z=-3.088; p=0.002). We also observed that physicians who were working in KASCH exhibited significantly better attitudes (Z=-2.018; p=0.044) but better practices were achieved by KAMC (Z=-2.712; p=0.007). Furthermore, the knowledge (H=6.320; p=0.042) and attitude (H=10.794; p=0.005) were better among consultants but residents demonstrated better practices (H=19.076; p<0.001). Likewise, physicians with radiology specialty exhibited better knowledge than other specialties (H=62.941; p<0.001). However, anesthesiologist physicians were found to have less with practices (H=57.017; p<0.001). Conversely, physicians who graduated in the EU/US demonstrated better knowledge (H=16.037; p<0.001) and attitude (H=12.988; p=0.002), however, physicians who graduated from Arab countries showed better practices (H=13.573; p=0.001). In addition, physicians with 11-20 years of clinical practice showed significantly better attitudes (H=21.108; p<0.001) while physicians with more than 20 years in clinical practice demonstrated poor practice (H=15.261; p=0.002).

**Table 5:** Differences in the score of the knowledge, attitude, and practices in regard to socio-demographic characteristics of the participants (n=454) KAMC – King Abdulaziz Medical City; KASCH – King Abdullah Specialist Children’s Hospital. ^a^ P-value has been calculated using Mann-Whitney Z-test. ^b^ P-value has been calculated using Kruskal Wallis H-test. ** Significant at p<0.05 level.

Factor	Knowledge Score (23) Mean ± SD	Attitude Score (3) Mean ± SD	Practices Score (8) Mean ± SD
Gender ^a^			
Male	10.0 ± 6.89	1.85 ± 1.07	5.36 ± 1.68
Female	7.88 ± 7.06	1.94 ± 1.04	5.53 ± 1.66
Z-test; p-value	-3.088; 0.002 **	-0.765; 0.444	-1.133; 0.257
Workplace ^a^			
KAMC	8.96 ± 6.95	1.83 ± 1.05	5.55 ± 1.70
KASCH	9.80 ± 7.23	2.03 ± 1.08	5.12 ± 1.54
Z-test; p-value	-1.163; 0.245	-2.018; 0.044 **	-2.712; 0.007 **
Occupation ^b^			
Consultant	10.1 ± 7.42	2.14 ± 1.02	4.99 ± 1.66
Resident	9.17 ± 6.81	1.77 ± 1.07	5.68 ± 1.67
Other	7.62 ± 7.04	1.88 ± 1.05	5.17 ± 1.47
H-test; p-value	6.320; 0.042 **	10.794; 0.005 **	19.076; <0.001 **
Specialty ^b^			
Surgery	9.91 ± 7.31	1.81 ± 1.09	5.79 ± 1.59
Medicine	8.32 ± 6.57	1.78 ± 1.11	5.91 ± 1.67
Pediatrics	11.9 ± 6.49	2.13 ± 0.95	5.43 ± 1.28
Gynecology	8.50 ± 6.99	1.75 ± 1.07	5.45 ± 1.64
Orthopedics	7.00 ± 6.34	1.89 ± 0.93	5.78 ± 1.72
Anesthesia	7.33 ± 6.12	1.79 ± 1.24	3.88 ± 1.65
Emergency	9.30 ± 6.41	1.62 ± 1.01	5.94 ± 1.73
Family medicine	6.47 ± 7.18	1.89 ± 1.00	4.81 ± 1.39
Radiology	15.9 ± 4.39	2.21 ± 1.05	5.09 ± 1.74
Others	7.04 ± 7.05	1.93 ± 1.07	5.67 ± 1.65
H-test; p-value	62.941; <0.001 **	12.356; 0.194	57.017; <0.001 **
Graduation place ^b^			
EU/US	13.3 ± 6.71	2.44 ± 0.97	4.94 ± 1.58
Arab country	8.87 ± 6.93	1.84 ± 1.05	5.53 ± 1.65
Other	8.20 ± 7.42	1.75 ± 1.21	4.30 ± 1.66
H-test; p-value	16.037; <0.001 **	12.988; 0.002 **	13.573; 0.001 **
Years of clinical practice ^b^			
<5 years	8.90 ± 6.79	1.79 ± 1.06	5.55 ± 1.71
5 – 10 years	9.24 ± 7.32	1.75 ± 1.14	5.59 ± 1.59
11 – 20 years	11.2 ± 6.78	2.46 ± 0.84	5.11 ± 1.34
>20 years	8.77 ± 7.94	2.18 ± 0.89	4.66 ± 1.69
H-test; p-value	5.893; 0.117	21.108; <0.001 **	15.261; 0.002 **

## Discussion

Based on our results, the majority of physicians showed poor knowledge, and practice, whereas their attitude was positive as they were willing to reduce the use of routine X-ray, fluoroscopy and CT scan. In fact, the poor knowledge aligns with two other regional studies conducted in Palestine and Egypt [19,20]. One interesting finding in our results was that male physicians had significantly better knowledge than female physicians. However, further studies should be conducted to investigate this gap.

The results varied between different specialties. Radiologists had the best knowledge regarding the hazards of radiation followed by pediatricians, while orthopedists had the least knowledge which was consistent with the literature [21]. Radiologists also scored the highest in attitude. Surprisingly, among the nine departments in which the questionnaire was distributed, the radiology department ranked seventh in the practice score. This gap between knowledge and practice among radiologists can be attributed to the fact that radiology training programs are rich in information about radiological hazards as it is a core in their study field, while poor practice among radiologists may be explained by the frequent exposure to radiation which makes them prone to neglect protection and safety measures even more.

Compared to another study that emphasized the prevalence locally in Saudi Arabia and included more than 450 physicians, our study had a larger sample size which gave a better insight into the level of awareness. In the previously mentioned study, about 73% of the participants had not received education about radiation protection [15]. In addition, their result showed that surgeons had the poorest knowledge. However, in our study, surgeons had the third highest score in terms of sufficient knowledge [15]. This also was contrary to another study that stated that surgeons were the most insufficient in terms of knowledge [22]. Since personal safety is a fundamental pillar in the medical field, the International Commission on Radiological Protection (ICRP) has recommended different protection measurements. One of which is the concept of dose limits that applies to the public as well as occupationally exposed workers. Yet, when it comes to medical application and diagnosing patients, there is no such limit as this may hinder proper health care delivery [23]. Unexpectedly, in our study, only 2% of the participants correctly identified the patient’s radiation dose limit, which is no limit, rendering it the least accurately answered question. Additionally, around 70% of physicians reported being unaware of As Low As Reasonably Achievable (ALARA) principle. Likewise, a similar response was observed in the Palestine study [19]. This is a shocking percentage since ALARA is considered the most basic concept of radiation safety that is even taught to the undergraduate students in medical schools nowadays.

Furthermore, approximately half of the participants in the Palestine study stated that they know published articles on radiation hazards, while in our study, only one-thirds of the participants did so [19]. On the contrary, a study conducted in Italy in 2017 revealed that the majority of participants had an excellent level of knowledge [24]. Additionally, the results of a study conducted in Iran showed an overall good radiation safety awareness among staff in 18 hospitals [25]. Moreover, one-third of the physicians in a previous study were unable to recognize the most sensitive organ. However, in our study, it was surprising that two-third of the physicians who participated were unable to identify the gonads as the most susceptible organ to ionizing radiation [15].

Moreover, about 40% of the physicians mentioned using routine X-ray more than 75% of the time. This overuse may be attributed to the fact that some patients insist on medical imaging and refuse to be clinically diagnosed. The lack of knowledge about the consequences of ionizing radiation and its financial cost might be a main contributor to the high demand for X-rays by patients. Therefore, focused education of the public and patients is necessary to avoid this conflict. Nevertheless, the overutilization of imaging can be a result of the physicians themselves as they might have little knowledge about alternatives that can provide, in some cases, the same results of ionizing imaging at less cost and which causes less harm to patients. Also, some physicians may request an X-ray without thoroughly doing a careful physical exam. Lastly, physicians can face difficulty in making a definitive diagnosis in some cases, therefore, the referring physician will request unnecessary imaging to get closer to pinpoint a diagnosis.

This research, however, is subject to several limitations. The first is that the study used a self-reported questionnaire, as the respondents may over-rate themselves, and this affects the accuracy of the results. Another factor that may have affected our data collection is that it was during the period of COVID-19 pandemic. Due to the pandemic, the survey was conducted online which makes it more likely for the respondents to misunderstand the questions since handing out the questionnaire face to face is helpful in case any unclear questions need to be interpreted to the respondents. In addition to the limitations, our study covered the National Guard hospital only which affects the generalizability of the results.

## Conclusions

Our participants revealed poor knowledge and practice while they had a positive attitude, and this varied between different specialties. Our results and similar results in the region pointed out that there is a huge gap in radiation safety awareness that needs to be encountered and further investigated. Additionally, further research should be done to increase the validity of the results and to accurately assess the level of awareness in physicians concerning the radiological hazards.

## Appendices

**Table 6:** Questionnaire

Section 1:
Workplace: King Abdulaziz Medical City, King Abdullah Specialist Children’s Hospital
Sex:
Male
Female
Occupation:
Consultant
Resident
Other
Specialty:
Surgery
Medicine
Paediatrics
Gynaecology
Orthopaedics
Anaesthesia
Emergency
Country of medical graduation:
European country/United States
Arab country
Former Soviet Union country
Other
Years of clinical practice:
< 5
5–10
11–20
>20

Section 2:
Are you familiar with ALARA principle:
Yes
No
Do you know any published articles on radiation hazards:
Yes
No
Do you know about FDA listing medical X-rays as a known carcinogen:
Yes
No
What do you think the radiation dose to patient from multi-slice CT scanner is:
Higher than single-slice helical scanner
Lower than single-slice helical scanner
Similar to single-slice helical scanner
Don’t know
% of total ionizing radiation the general public is exposed to from medical radiation:
1–10
15–30
35–45
60–75
80–95
Don’t know
What is the recommended patient dose limit for medical radiation (mSv):
100
50
20
5
0.5
No dose limit
Don’t know
What are ICRP recommendations for professional responsibility for protecting patients:
According to the freedom of prescription
Prescriber, not practitioner
Practitioner, not prescriber
Both prescriber and practitioner
Don’t know

Estimated sensitivity level	Lungs	Bladder	Gonads	Kidneys	Stomach
	1	1	1	1	1
	2	2	2	2	2
	3	3	3	3	3
	4	4	4	4	4
	Don’t know	Don't know	Don’t know	Don’t know	Don't know

No. of chest X-ray equivalents	Lumbar spine X-ray	Abdominal CT scan	Barium enema
<1	<1	<1	<1
10	10	10	10
65	65	65	65
120	120	120	120
250	250	250	250
> 250	> 250	> 250	> 250
Don’t know	Don’t know	Don’t know	Don’t know

Frequency of requests	Never	Rarely (< 25% of the time)	Sometimes (25%–75% of the time)	Often (> 75% of the time)
Routine X-ray				
CT scan				

Will you reduce the use of the following method	Yes	No
Routine X-ray		
Fluoroscopy		
CT scan		
